# Rose Stem Cell-Derived Exosome-Hyaluronic Acid Therapy for Recurrent Atrophic Vaginitis: A Case Report

**DOI:** 10.7759/cureus.109973

**Published:** 2026-05-31

**Authors:** Marwa H Elajami

**Affiliations:** 1 Gynecology Department, CosmeSurge Group, Dubai, ARE

**Keywords:** atrophic vaginitis, exosomes, gsm, hyaluronic acid, non-hormonal therapy, vaginal regeneration

## Abstract

Atrophic vaginitis (AV), which is an aspect of genitourinary syndrome of menopause (GSM), is caused by low levels of estrogen, which leads to weakening of the vaginal lining, vaginal dryness, vaginal irritation, and high vaginal pH. The side effects related to long-term use of hormonal therapies are the primary reason for the search for non-hormonal therapeutic options. In this case report, a 41-year-old perimenopausal female presented with recurrent vaginal discomfort, vaginal dryness, pruritus, and occasional offensive vaginal discharge that had not responded to repeated antimicrobial therapy. The clinical examination revealed that the mucosa was pale and thinned, without signs of infection, and lactobacilli levels were reduced. The first biopsy revealed benign, mature squamous mucosa with mild atrophy, focal basal cell hyperplasia, focal glycogen deposition, and fibrous stroma. She underwent three sessions of stem cell exosomes-hyaluronic acid at 3- to 4-week intervals. The intervention consisted of rose stem cell-derived exosomes supplied as a lyophilized freeze-dried powder, reconstituted with hyaluronic acid solution before administration. The product contained approximately 3 billion exosomes in a 2.5 mL vial, with a reported nanoparticle size range of 50-100 nm and purity above 90%. The preparation was stored at 2-8 °C and administered intravaginally using electroporation followed by topical application. Follow-up biopsy demonstrated a categorical increase in epithelial thickness (approximately 2.5 mm), retained maturation, slight residual atrophy, no glycogen on PAS-D, or abnormal fibrovascular stroma, and no dysplasia. The patient reported symptomatic relief, and follow-up assessment showed partial structural mucosal changes without dysplasia or malignancy on short-term biopsy. No short-term local adverse events, such as irritation, bleeding, infection, hypersensitivity, or worsening discharge, were reported during the available follow-up. However, this single uncontrolled case cannot establish long-term safety or treatment efficacy.

## Introduction

Atrophic vaginitis (AV), also known as vulvovaginal atrophy (VVA), is most commonly described in postmenopausal women, but similar estrogen-deficient vulvovaginal changes may also occur during the perimenopausal or climacteric transition [1-3]. About 45% of postmenopausal women develop symptoms that can be related to AV [4]. These symptoms are vaginal dryness, dyspareunia, pruritus, vaginal PH changes, and several urinary and genital complaints. Its underlying pathogenesis is a decrease in circulating estrogen levels following menopause, which predisposes to gradual changes in the physiology and structure of the vulvovaginal mucosa [4].

Although most epidemiological and therapeutic literature on VVA and genitourinary syndrome of menopause (GSM) focuses on postmenopausal women, the pathophysiological process may begin during the menopausal transition. Perimenopausal and climacteric women can experience fluctuating and declining estrogen levels, leading to reduced epithelial maturation, decreased lubrication, elevated vaginal pH, altered lactobacilli predominance, and symptoms such as dryness, irritation, dyspareunia, and recurrent vaginal discomfort. Therefore, postmenopausal literature is relevant to the present case because it describes the same estrogen-deficient mucosal pathway; however, its applicability to a perimenopausal patient should be interpreted cautiously because the degree and stability of hypoestrogenism may differ from established menopause.

Hypoestrogenism status cannot only undermine the integrity of the vaginal epithelium, but a poor quality of life and predisposition to secondary complications in the form of frequent UTI may also occur [5-7]. Although the menopausal transition presents mild genital alterations in most women, no less than 10-47 % will experience moderate to severe debilitating symptoms such as dryness, dyspareunia, and pain in the vulva, frequent urinary infections, and altered vaginal discharge [8].

Even though topical estrogen therapy is the most effective intervention to reverse the mucosal atrophy, non-hormonal treatment options are of utmost significance. Many women are reluctant to take estrogen on the grounds of potential side effects, and others have absolute or relative contraindications of hormonal treatment. As a result, a significant number of patients choose non-hormonal methods to treat their symptoms [9]. Furthermore, vaginal estrogen treatment requires patient adherence. A patient preference trial reported that, despite an overall treatment duration of eight weeks, compliance was limited by inconvenience and vaginal discharge, suggesting that increasing the treatment duration (>3 months) could improve compliance and lead to suboptimal adherence [10].

In this context, the present case report describes a non-hormonal therapeutic option for AV, addressing the need for effective alternatives for women seeking estrogen-free management strategies.

## Case presentation

A 41-year-old, perimenopausal multiparous female presented with recurrent vaginal discomfort, including vaginal dryness, vaginal pruritus, and occasional offensive vaginal discharge. These symptoms persisted even after several courses of empirical and vaginal swab culture-based antimicrobial treatment. Clinical examination revealed features consistent with AV, prompting further microbiological, cytological, vaginoscopic, hormonal, ultrasonographic, and histopathological assessment to support the diagnosis and exclude common infectious, inflammatory, dermatological, and neoplastic mimics.

An extensive assessment was conducted to determine the recurrence of vaginitis and assess underlying mucosal health. The clinical examination revealed thin, weakened squamous epithelium, dryness, but no lesions, ulcerations, or suspicious neoplastic stigmas by the vaginoscope. Vaginal pH was high (> 5.0), and vaginal smears' microscopic analysis revealed low lactobacilli counts and the presence of parabasal cells, which is typical of estrogen-deficient atrophic physiology; microcultures and sexually transmitted infection (STI) screening did not show any pathogenic growth. Pelvic ultrasound revealed a normal uterus and adnexa, endometrial thickness of a luteal phase, and hormonal evaluation showed that the patient had a perimenopausal and climacteric state and no absolute ovarian insufficiency. This hormonal profile was considered clinically relevant because the patient’s symptoms, elevated vaginal pH, reduced lactobacilli, presence of parabasal cells, pale thinned mucosa, and biopsy findings were consistent with hypoestrogenic mucosal changes despite the absence of complete ovarian insufficiency. To further characterize mucosal integrity and exclude dysplasia in the setting of persistent symptoms, a detailed histopathological evaluation was undertaken.

Differential diagnosis and exclusion of alternative causes

Given the long history of recurrent vaginal dryness and vaginitis-like symptoms, alternative causes of chronic vulvovaginal irritation were considered before attributing the symptoms to AV. Repeated vaginal swabs and cytological evaluations did not demonstrate pathogenic bacterial and fungal infections or STIs. Desquamative inflammatory vaginitis was considered less likely because there was no marked purulent discharge, no prominent inflammatory cytology, and no dense inflammatory infiltrate on biopsy. Contact dermatitis and allergic vulvovaginitis were considered clinically unlikely because the patient did not report a temporal relationship with irritants, topical agents, hygiene products, or medications, and examination did not reveal eczematous vulvar changes. Vulvodynia was also considered unlikely because the predominant symptoms were dryness, pruritus, and recurrent irritation rather than isolated vulvar pain or allodynia. Lichen sclerosus and other inflammatory vulvar dermatoses were not supported clinically because there were no ivory-white plaques, architectural distortion, fissuring, scarring, erosive lesions, or suspicious vulvar skin changes. Histopathological examination showed benign squamous mucosa with mild atrophic changes and no dysplasia, malignancy, or specific inflammatory dermatosis. Therefore, the overall clinical, microbiological, cytological, vaginoscopic, and histopathological findings supported estrogen-deficient AV as the most likely diagnosis (Table 1).

**Table 1:** Differential diagnosis considered in recurrent vaginal symptoms

Differential diagnosis	Reason considered	Findings arguing against diagnosis
Recurrent infectious vaginitis	Recurrent discharge, pruritus, and irritation	Repeated vaginal swabs and STI screening did not show pathogenic growth
Desquamative inflammatory vaginitis	Chronic irritation, elevated pH, and discharge may overlap	No marked purulent discharge, no prominent inflammatory cytology, and no dense inflammatory infiltrate on biopsy
Contact dermatitis/allergic vulvovaginitis	Chronic irritation and pruritus may mimic vaginitis	No clear irritant exposure history and no eczematous vulvar changes on examination
Vulvodynia	Chronic vulvovaginal discomfort	Symptoms were dominated by dryness, pruritus, mucosal pallor, and atrophic changes rather than isolated vulvar pain/allodynia
Lichen sclerosus	Chronic vulvar irritation and pruritus	No ivory-white plaques, scarring, fissuring, architectural distortion, or typical vulvar dermatosis
Other inflammatory dermatoses	Can cause chronic irritation and mucosal symptoms	No erosive, ulcerative, eczematous, or specific inflammatory lesions; biopsy did not show a specific dermatosis
Dysplasia/neoplasia	Persistent symptoms required exclusion of premalignant/malignant disease	Biopsy showed no dysplasia or malignancy
Atrophic vaginitis / estrogen-deficient mucosal change	Dryness, high pH, reduced lactobacilli, parabasal cells, and pale, thinned mucosa	Supported by clinical examination, cytology, pH, reduced lactobacilli, and biopsy showing mild atrophic changes

**Figure 1:**
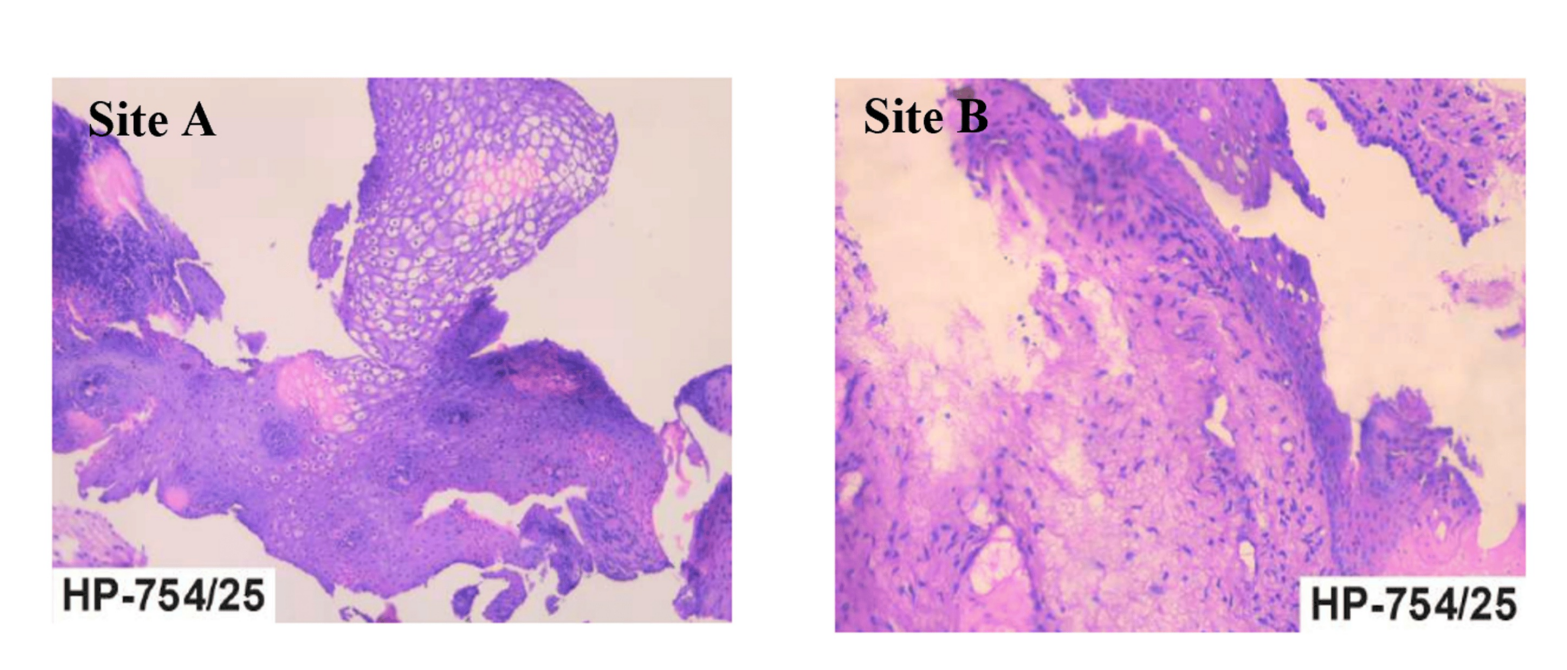
Histopathology of the first biopsy Site A (Upper Vagina). Slide 25MLH20254 shows benign squamous mucosa with maturation (thickness approx. 2.0 mm), focal basal cell hyperplasia, and mild atrophy with fibrous stroma and minimal chronic inflammation. No dysplasia or neoplasia identified. PAS stain shows focal glycogen deposits. The trichrome stain reveals fibrous tissue measuring approximately 1.00 mm. No hyperplasia or neoplasia identified. Written informed consent was obtained from the patient for publication of anonymized clinical images. Site B (Lower Vagina). Slide 25MLH20254 shows the same of the upper vaginal biopsy histopathology; benign squamous mucosa with maturation (thickness approx. 2.0 mm), focal basal cell hyperplasia, and mild atrophy with fibrous stroma and minimal chronic inflammation. No dysplasia or neoplasia identified. PAS stain shows focal glycogen deposits. The trichrome stain reveals fibrous tissue measuring approximately 1.00 mm. No hyperplasia or neoplasia identified.

The initial biopsy was conducted when she had recurrent vaginitis, and the vaginal mucosa appeared clinically pale, showing atrophic alterations and loss of vaginal rugosities. Two sites were sampled, including Site A (Upper Vagina) and Site B (Lower Vagina). The laboratory received five slides and two blocks, including H&E-stained sections and PAS- and trichrome-stained sections for second-opinion evaluation. The biopsy revealed benign squamous mucosa approximately 2.0 mm thick, with focal atrophy, basal cell hyperplasia, minimal chronic inflammation, minimal focal glycogen deposits on PAS stain, and a fibrous stroma, as indicated by trichrome staining (Figure 1).

Epithelial thickness was assessed histopathologically on well-oriented H&E-stained sections by measuring the vertical distance from the basal epithelial layer to the superficial epithelial surface in the most representative non-tangential areas. The biopsy was obtained from the same anatomical vaginal region by the same treating physician, and the histopathological assessment was performed by the same histopathologist to minimize inter-observer and sampling variability.

Based on the clinical and histopathological findings, the patient was treated with rose stem cell-derived exosome-hyaluronic acid therapy. The exosome product was manufactured by ExoCoBio/BENEV (California, US) and supplied as a lyophilized freeze-dried powder, which was dissolved in hyaluronic acid solution immediately before use. According to the product specifications, each vial contained approximately 3 billion exosomes in 2.5 mL, with reported nanoparticle size ranging from 50 to 100 nm and purity above 90%. The product was stored at 2-8°C until administration. Hyaluronic acid in this preparation was used as a high-molecular-weight carrier and hydrating agent, intended to improve mucosal hydration and lubrication; therefore, any clinical improvement cannot be attributed to exosomes alone. The route of administration was intravaginal; electroporation was first performed to facilitate mucosal uptake, followed by the topical application of the exosome-hyaluronic acid preparation evenly over the vaginal walls under direct visualization. Three treatment sessions were performed at three- to four-week intervals. The patient was monitored after each session for local irritation, pain, bleeding, abnormal discharge, infection, allergic reaction, or other adverse events.

Before treatment, the patient was counselled regarding the nature of rose stem cell-derived exosome-hyaluronic acid therapy, its intended non-hormonal regenerative use, the limited clinical evidence available for this indication, possible benefits, potential risks, and alternative treatment options, including conventional non-hormonal and hormonal therapies. Written informed consent was obtained for the intervention, clinical examination, biopsy procedures, follow-up evaluation, and use of anonymized clinical, vaginoscopic, and histopathological images for scientific publication. The product was used as a commercially available exosome-hyaluronic acid preparation; however, its regulatory approval status for AV should be interpreted according to the applicable local regulatory framework. No patient-identifying information has been included in the manuscript.

**Figure 2:**
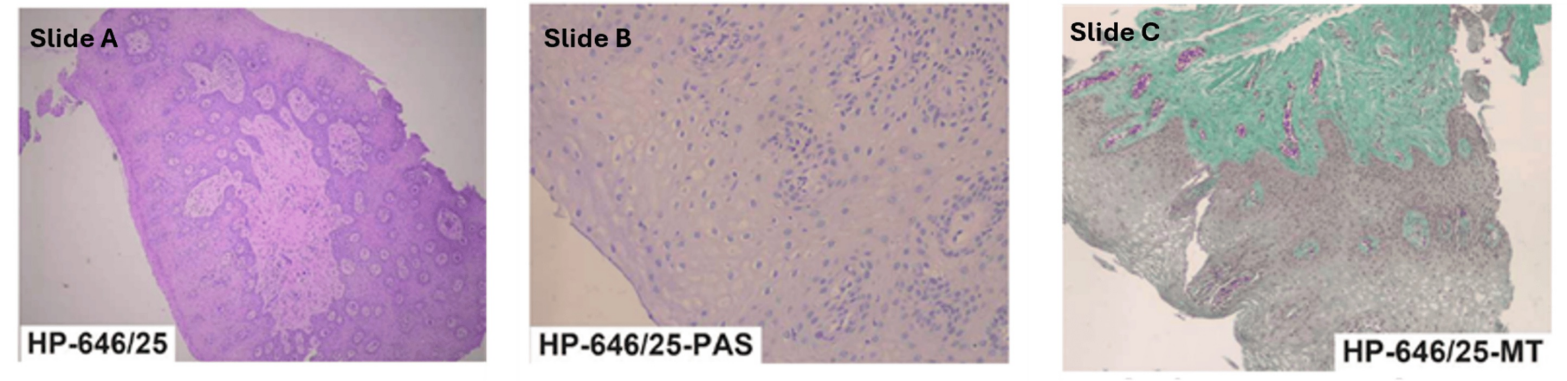
Second biopsy of the vagina Slide A: H&E stain shows benign mature squamous mucosa composed of a basal layer and multiple layers of intermediate and superficial squamous cells (with a thickness of approximately 2.5 mm). Slide B: Trichome stain shows a minimal focus of atrophy and basal cell hyperplasia, and normal underlying fibrovascular tissue. Slide C: The PAS/D stain did not show glycogen deposits. No dysplasia or malignancy was identified. Written informed consent was obtained from the patient for publication of anonymized clinical images.

The follow-up biopsy was performed one month after the third and final treatment session (Table 2). The patient had received three sessions of rose stem cell-derived exosome-hyaluronic acid therapy at three to four-week intervals after the baseline biopsy. The specimen was placed in a single cassette containing formalin, which consisted of several pieces of soft tissue of short height, measuring around 0.4 x 0.3 x 0.1 cm, all in one container. PAS and trichrome staining were performed. This specimen exhibited benign, mature squamous mucosa with increased epithelial thickness (approximately 2.5 mm), a minimal residual focus of atrophy, mild basal cell hyperplasia, and a normal fibrovascular stroma. PAS-D staining revealed no glycogen deposits, dysplasia, or malignancy (Figure 2).

**Table 2:** Details of the rose stem cell-derived exosome–hyaluronic acid intervention

Parameter	Details
Exosome source	Rose stem cell-derived exosomes
Manufacturer	ExoCoBio/BENEV
Product form	Lyophilized freeze-dried powder
Reconstitution medium	Hyaluronic acid solution
Purity	Above 90%
Concentration	Approximately 3 billion exosomes per vial
Vial volume	2.5 mL
Nanoparticle size	50–100 nm
Storage condition	2–8°C
Route of administration	Intravaginal
Administration method	Electroporation followed by topical application
Application site	Even application over the vaginal walls
Number of sessions	Three sessions
Treatment interval	Every 3–4 weeks
Safety monitoring	Pain, irritation, bleeding, discharge, infection, allergic reaction, or other adverse events

The follow-up biopsy was obtained from the same anatomical vaginal region as the initial biopsy and processed using the same histopathological approach. The reported increase in epithelial thickness from approximately 2.0 mm to 2.5 mm was therefore interpreted as a supportive structural observation rather than a stand-alone endpoint. Because tissue orientation, fixation, sectioning, and processing may influence histological thickness measurements, this finding was considered together with the patient’s clinical improvement, including the disappearance of dryness, pruritus, and recurrent irritation after the treatment sessions.

**Figure 3:**
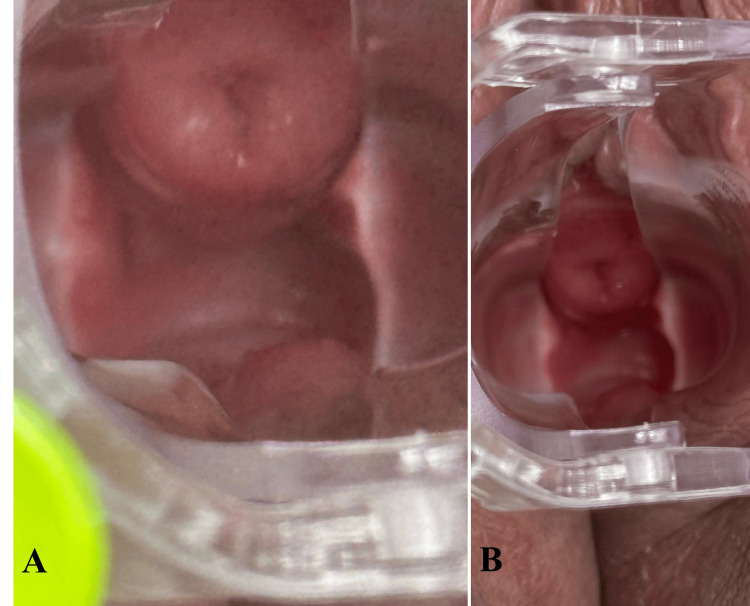
Vaginal mucosa clinical examination pre–and post-exosome-hyaluronic acid therapy A: Pretreatment vaginoscopic picture of pale vaginal mucosa with a lack of surface moisture and lightened color, which is related to the decrease in hydration and vascular perfusion with atrophic alterations. B: Three-session post-treatment image of the exosome-hyaluronic acid therapy that shows a better mucosal coloration and more surface moisture, which implies better hydration and blood perfusion.

The results of the clinical examination corroborated the histopathological improvements seen at the follow-up biopsy stage. Before treatment, vaginoscopic analysis showed pale vaginal mucosa with low moisture and a light surface color, characteristic of reduced hydration and poor vascularity. Following three sessions of exosome-hyaluronic acid therapy, re-examination showed clearly increased mucosal humidity and a more pink color, indicative of increased hydration and increased blood flow to the vaginal lining (Figure 3). These clinical observations were in sync with the changes observed in the second biopsy.

Although the patient reported symptomatic improvement after treatment, standardized pre- and post-treatment outcome measures such as the Vaginal Health Index, Vaginal Maturation Index, or validated symptom severity scores were not prospectively recorded. Therefore, the clinical response was assessed descriptively based on patient-reported symptom relief, vaginal pH/smear findings, vaginoscopic appearance, and comparative histopathological assessment.

A comparative analysis of the two biopsies (Table 3) revealed a measurable improvement in epithelial thickness and a reduction in atrophic changes following therapy, indicating a positive clinical and histological outcome of the exosome-hyaluronic acid treatment.

**Table 3:** Comparative clinical and histopathological findings before and after exosome-hyaluronic acid therapy, showing partial mucosal structural improvement with persistent uncertainty regarding glycogen restoration

Parameter	Biopsy I (Before treatment)	Biopsy II (After treatment)
Clinical signs		
Vaginal dryness	Present	Absent
Pale vaginal mucosa	Present	Absent
Reduced surface moisture	Present	Absent
Diminished mucosal perfusion (color)	Present	Absent
Loss of vaginal rugosities	Present	Marked improvement
Histopathological Findings		
Epithelial thickness	~2.0 mm	~2.5 mm
Epithelial maturation	Mature squamous epithelium with mild atrophy	Mature squamous epithelium with a minimal residual focus of atrophy
Basal cell hyperplasia	Present, focal	present, mild
Glycogenation (PAS / PAS-D)	Focal glycogen deposits are present	Glycogen deposits not identified
Inflammation	Minimal chronic inflammation	No significant inflammation reported
Subepithelial stroma	Fibrous stroma (~1.0 mm), highlighted by trichrome	Normal fibrovascular stroma, highlighted by trichrome
Dysplasia / neoplasia	None	None

## Discussion

This case illustrates recurrent AV in a perimenopausal woman with persistent symptoms despite antimicrobial therapy. Sequential biopsies obtained from comparable adjacent vaginal mucosal areas showed certain structural changes after exosome-hyaluronic acid therapy, including increased epithelial thickness, retained squamous maturation, and reduced atrophic foci. However, the absence of glycogen deposits on follow-up PAS-D staining indicates that complete functional restoration of the vaginal epithelium cannot be concluded. Therefore, the histological findings should be interpreted as partial mucosal structural improvement rather than definitive reversal of AV.

A key contextual issue in this case is that the patient was perimenopausal rather than definitively postmenopausal. Most available evidence regarding VVA, GSM, estrogen therapy, hyaluronic acid, and regenerative interventions has been generated in postmenopausal populations. Nevertheless, the cited literature remains biologically relevant because the patient demonstrated clinical, microbiological, and histopathological features compatible with estrogen-deficient vaginal mucosal change, including dryness, elevated vaginal pH, reduced lactobacilli, parabasal cells, mucosal pallor, epithelial thinning, and mild atrophic changes on biopsy. Therefore, the postmenopausal literature was used to support the underlying pathophysiological framework, while the findings of this case were interpreted cautiously because perimenopausal mucosal changes may fluctuate over time.

VVA occurs in between a quarter and a half of the postmenopausal women [11]. Various names, such as vaginal atrophy, AV, and urogenital atrophy, are applied interchangeably to describe the symptom complex associated with decreased estrogenization of the vaginal vulva and vaginal tissues [12]. Being a key aspect of GSM, estrogen deficiency causes the thinning of vaginal epithelium, a decrease in lubrication, a decrease in blood flow, an increase in pH, and increased vulnerability to dry and painful intercourse. All of these factors lead to sexual dysfunction and low quality of life among peri- and postmenopausal women [13,14].

Even though systemic and topical estrogen treatments can ameliorate stress and rehabilitate epithelial thickness, the issue of breast and endometrial cancer or cardiovascular risk usually restricts their use over time [15,16]. Symptoms of VVA remain prominent in many postmenopausal women even after menopausal hormone therapy (MHT), and there have been safety concerns with long-term use [11]. Symptomatic relief may be achieved with non-hormonal agents, such as lubricants and moisturizers, but they have little effect in reversing underlying histological atrophy [17]. Consequently, numerous women, in particular, those who are sceptical of hormone exposure or who have contraindications, prefer non-hormonal methods of treatment [9].

Emerging regenerative therapies offer promising alternatives. In vitro experiments show that stem cell-derived exosomes can stimulate VK2 vaginal epithelial cell proliferation and migration, increase the expression of Ki67 and CD31, and significantly increase epithelial thickness in an exosome hydrogel [9]. The carrier, hyaluronic acid (HA) in this instance, has the added advantage of being proven to support hydration, lubrication, and the integrity of the extracellular matrix. Dos Santos et al. (2021) concluded that, in their systematic review, HA was as effective as vaginal estrogen in reducing symptoms of dryness, dyspareunia, high pH, and cell maturation [18]. Recent results also favor HA as a safe, effective first-line treatment, particularly in mild cases of vaginal atrophy [19].

In this case, the combined use of exosomes and HA led to quantifiable epithelial regeneration, characterized by increased mucosal thickness and reduced atrophic changes. These developments align with the complementary mechanisms of both components, as exosomes drive cellular repair and HA supports hydration and structural integrity. The absence of dysplasia or neoplasia on short-term follow-up biopsy is reassuring but does not establish clinical safety. In this case, no short-term local adverse events, such as irritation, bleeding, infection, hypersensitivity, or symptom worsening, were reported; however, systematic adverse-event monitoring over longer follow-up is required before safety can be assessed.

The detailed reporting of the product source, manufacturer, concentration, particle size, purity, storage conditions, and administration route is important because exosome-based interventions may vary substantially according to source material, preparation method, particle concentration, and quality-control standards (Table 1).

The findings of this case report should be interpreted cautiously because this is a single uncontrolled observation, and a causal relationship between exosome-hyaluronic acid therapy and the observed clinical or histological changes cannot be established. The apparent improvement may have been influenced by sampling variation, natural fluctuation of perimenopausal mucosal status, observer interpretation, placebo effect, or the independent effect of hyaluronic acid. Although the follow-up biopsy was obtained from an adjacent area to the initial biopsy site, it was not taken from the same microscopic location. In addition, most comparative literature relates to postmenopausal GSM/VVA, whereas this patient was perimenopausal, limiting direct extrapolation. Larger controlled studies using standardized biopsy sites, validated symptom scores, vaginal pH, maturation index, and objective glycogen assessment are required before definitive conclusions can be drawn. Another limitation is the absence of validated pre- and post-treatment clinical endpoints, such as a standardized symptom score, Vaginal Health Index, or Vaginal Maturation Index, which limits objective quantification of the degree of improvement.

## Conclusions

This case report describes a 41-year-old perimenopausal woman with recurrent atrophic vaginitis-like symptoms who showed symptomatic relief and partial structural mucosal changes after rose stem cell-derived exosome-hyaluronic acid therapy. The follow-up biopsy, taken from an area adjacent to the initial biopsy site, demonstrated increased epithelial thickness and reduced atrophic changes; however, PAS-D staining did not show glycogen restoration. Therefore, these findings should be interpreted with caution and not considered definitive evidence of complete epithelial functional recovery or treatment efficacy. The observed improvement is encouraging and suggests that exosome-hyaluronic acid therapy may represent a potential non-hormonal therapeutic option for selected patients who are unwilling or unsuitable for estrogen-based therapy. However, because this is a single uncontrolled case and validated outcome measures such as symptom severity scores, Vaginal Health Index, or Vaginal Maturation Index were not prospectively recorded, the degree of clinical improvement cannot be objectively quantified. Larger controlled studies with standardized clinical endpoints, objective histological assessment, and longer follow-up are required before firm conclusions regarding efficacy and safety can be made.
